# A systematic review of patient-oriented outcomes following complete denture treatment: a comparison between the neutral zone technique and conventional approach

**DOI:** 10.1038/s41405-024-00222-7

**Published:** 2024-05-23

**Authors:** Nareudee Limpuangthip, Siraphob Techapiroontong, Wisarut Prawatvatchara

**Affiliations:** Department of Prosthodontics, Faculty of Dentistry, Chulalongkorn University, Bangkok, 10330 Thailand

**Keywords:** Prosthetic dentistry, Dental treatments

## Abstract

**Objectives:**

To determine the patient-oriented outcomes after complete denture (CD) treatment using neutral zone (NZ) techniques compared with those of conventional dentures.

**Materials and methods:**

Electronic and hand searches were conducted up to December 2023 based on PICOS criteria. Population (P) was patients with complete edentulism on maxillary and mandibular arches and were either or not wearing CDs. Intervention (I) focused on the fabrication of mandibular and/or maxillary CD using NZ techniques. Comparators (C) included other CD fabrication approaches, such as conventional and simplified techniques, and the use of old or existing CDs. Outcomes (O) were patient-oriented treatment outcomes. Study design (S) included human studies.

**Results:**

Eleven human experimental studies were included. NZ dentures demonstrated better patient-reported outcomes, by providing greater comfort, enhancing denture stability and retention, reducing food traps underneath the denture, as well as improving appearance, chewing efficiency and speech. Objective findings varied, with most studies showing equivalent outcomes for NZ and conventional dentures. However, one study indicated superior, and another demonstrated worse outcomes for NZ dentures.

**Conclusions:**

NZ dentures generally improve patient-reported outcomes more than conventional dentures. However, their impact on objective outcomes compared with a conventional denture remains uncertain.

## Introduction

Fabricating complete dentures (CD) for patients with atrophic residual alveolar ridges poses a significant challenge because their physiologic and anatomical limitations can hinder the creation of well-fitting dentures with proper contours and tooth arrangement [1]. This can result in difficulties achieving the desired prosthetic stability, comfort, and function [2, 3]. One critical concept for this context is the neutral zone (NZ), which refers to the specific space within the oral cavity where the inward forces exerted by the lips and cheeks counterbalance the outward forces exerted by the tongue during various oral functions [4]. The NZ is also known by other various terms, such as the dead space [5], the stable zone [6], the zone of least interference [7], and the denture space [8].

To record the NZ during CD fabrication, several techniques have been reported in the literatures. These methods include the denture space recording, the myodynamic approach [9], the NZ [10] and the modified NZ techniques [11–13], as well as the flange technique [14], the piezograph [15], the muscle-formed complete mandibular denture [16], and the border molding [17]. The techniques are typically used in mandibular CD fabrication, especially for patients who exhibit severe mandibular ridge resorption and often cannot receive implant-retained prostheses due to physical, psychological, or financial limitations [11, 12].

To assess the outcomes of dental prosthodontic treatments, the patient-oriented outcomes are commonly used, encompassing objective and subjective measures [2, 3, 18, 19]. The objective measures involve professional evaluations of oral conditions, such as soft tissue quality, masticatory function, and speech production [11, 12, 20–22]. The subjective measures capture patient-reported outcomes, including satisfaction [9, 23, 24], perceptions [9, 13, 23, 25], and oral health-related quality of life (OHRQoL) [11, 26–28]. The OHRQoL is a multidimensional construct used to assess individual perception of their oral health and its impact on their quality of life, covering physical and psychosocial well-being [29].

Various clinical studies have assessed the efficacy of CD fabrication using the NZ techniques compared with a conventional approach [9, 11–13, 16, 20–24, 26, 28]. However, it is unclear whether the two treatment approaches lead to distinct outcomes. The intricate nature of clinical and laboratory procedures using NZ techniques makes it challenging to ascertain the suitability of the NZ technique for routine clinical application. Thus, the objective of this systematic review was to assess the patient-oriented treatment outcomes, including the objective outcomes and patient-reported outcomes, in a CD fabrication using NZ techniques compared with a conventional approach.

## Materials and methods

This study was conducted following the PRISMA (Preferred Reporting Items for Systematic Reviews and Meta-Analysis) statement [30]. The research question was “Does complete denture fabrication using NZ techniques provide better patient-oriented treatment outcomes compared with a conventional approach?” The protocol for this systematic review was registered with PROSPERO (International Prospective Register of Systematic Reviews): no. CRD42023464420.

### Eligible criteria for the included studies

The eligible criteria for the included studies were human studies that compared the treatment outcome of mandibular and/or maxillary CD fabricated using NZ techniques with conventional or simplified techniques. Selection of the included studies was based on PICOS criteria. Population (P) encompassed patients who presented with complete edentulism on either or both maxillary and mandibular arches and were either currently wearing or not wearing CDs. Patients with maxillofacial defects were not included. Intervention (I) was focused on the fabrication of mandibular and/or maxillary CD using any of the NZ or the denture space recording approaches, such as the NZ and its modifications, the piezography, and the myodynamic techniques. Comparators (C) included the CD fabricated with the conventional approach, or old/existing CDs. Outcomes (O) were patient-oriented treatment outcomes, divided into three categories: patients’ oral conditions, objective outcomes, and subjective or patient-reported outcomes. Study designs (S) included observational and experimental human studies.

### Information sources and search strategy

Literature searches were conducted using two strategies: electronic and manual searches. An electronic search was performed using the PubMed and SCOPUS databases, and the Cochrane Database of Systematic Reviews databases up to December 2023. The electronic search approach included electronic Medical Subject Headings [MeSH] search terms and keyword terms: (“dead space” OR “denture space” OR “neutral zone” OR piezograph* OR “flange technique” OR “muscle form” OR “zone of minimal conflict” OR “myodynamic” OR “stable zone”) AND (complete denture [MesH] OR denture). The manual search was based on the references to the identified articles. The search was restricted to human studies without language restrictions.

All titles and abstracts were screened, and the retrieved articles were individually reviewed for their eligibility criteria by N.L. and S.T. Any discrepancies were discussed and resolved by W.P. The excluded articles were letters to the editor, editorial commentaries, case reports and case series, narrative and systematic reviews, and studies with implant-retained overdentures.

### Data extraction

Data were independently extracted by two authors (N.L. and S.T.), and any disagreement was resolved by the third reviewer (W.P.). The extracted information comprised first author’s name and year of publication, study design, experimental and control groups together with sample size, characteristics of the participants, previous denture experience, denture provider, and descriptions of the NZ or denture space recording techniques. In addition, a summary was provided for the methods employed in both objective and subjective outcomes, along with details about the timing of evaluations and the preferred impression technique.

### Risk of bias assessment

The quality of the included clinical trials was assessed according to the Cochrane Handbook (version 6.4, 2023), using the Cochrane Risk of Bias (RoB) tool for randomized crossover trial studies [31], and the Risk Of Bias In Non-randomized Studies of Interventions (ROBINS-I) tool for non-randomized studies of interventions [32]. The evaluation was performed independently by N.L. and S.T., and any discrepancies were adjudicated by the third investigator (W.P.). Each domain received a three-level response: low, some concern, and high risk of bias.

## Results

### Study selection and characteristics

**Fig. 1:**
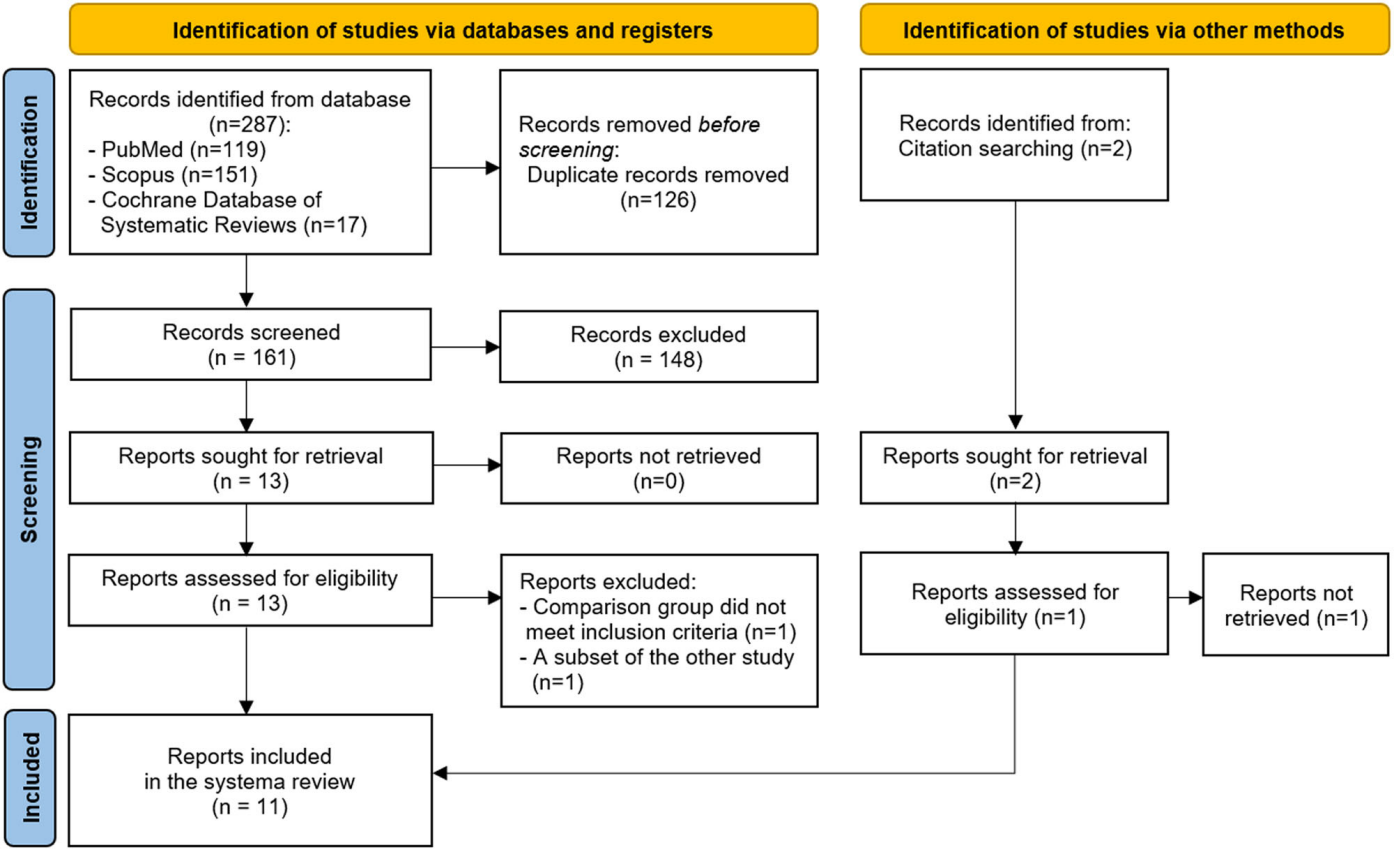
PRISMA flow diagram of article review and selection process.

A flow diagram of the selection process for the articles is presented in Fig. 1. Thirteen articles remained for a full-text review, and two studies were excluded after the full-text review [25, 26]. One of the excluded studies included a comparison group that was not a denture fabricated with conventional techniques [25], while the other study was considered a subset of the included study, presenting identical outcomes and findings but a smaller sample size [26, 28]. Finally, the present systematic review included eleven experimental studies, consisting of six crossover trial studies, and five quasi-experimental studies (Table 1). Four included studies involved the same samples from the same two settings [11, 12, 22, 23]. Several NZ techniques were included, comprising the muscle-formed complete mandibular denture [16], the myodynamic [9], the NZ [20, 22, 23], the piezography or phonetic NZ [22, 23], the swallowing NZ, and the modified NZ techniques [11–13].

**Table 1:** Characteristics of the included studies.

First author (Year)	Study design	Experiment & Control group (n/group)	Participants			Previous denture experience (Yes, No)	Denture provider(s)	Neutral zone (Denture space) recording technique			
			Total N	Sex (M:F ratio), Age (years)	Edentulous condition			Impression technique	Impression material	Material for polished surface record	Occlusal scheme
Walsh & Walsh (1976) [16]	Quasi-experiment (before and after)	Before (existing CV) & After (new muscle-formed) (*n* = 30)	30	N/A	N/A	Yes	N/A	Muscle-formed complete mandibular denture	Polysulfide/polyvinyl siloxane	Mouth-temperature wax	Free-sliding occlusion
Barrenas & Odman (1989) [9]	Randomized crossover trial	1) NZ-CV (*n* = 15) 2) CV-NZ (*n* = 15)	30	M:F = 1:1.14, mean=63.4 ± 8.5 y (33–78 y)	Slight, moderate, and severe ridge resorption	Yes	1 dentist	Myodynamic	Polyvinylsiloxane	Polyvinylsiloxane	N/A
Fahmy & Kharat (1990) [20]	Randomized crossover trial	1) NZ-CV (*n* = 10) 2) CV-NZ (*n* = 10)	10	N/A	N/A	No	N/A	NZ by Beresin & Schiesser (1976) [10]	Modeling plastic impression compound	ZOE impression paste	Bilateral balanced articulation
Al-Magaleh et al. (2012) [21]	Quasi-experiment (before and after)	1) Dentate (*n* = 10) 2) CV-NZ (*n* = 10)	20	M:F = 1.5:1, mean=52 y	Class I max-man relation, adequate interarch distance, normal tongue behavior and size	N/A	N/A	NZ	Tissue conditioning material	Tissue conditioning material	N/A
Rehmann et al. (2012) [13]	Quasi-experiment (before and after)	Before (existing CV) & After (new NZ) (*n* = 5)	5	mean=61 y	N/A	Yes	N/A	Modified NZ	Thermoplastic denture adhesive	Thermoplastic denture adhesive	Bilateral balanced articulation
Ladha et al. (2013) [22]	Randomized crossover trial	1) old CV-SNZ-PNZ (*n* = 5) 2) old CV-PNZ-SNZ (*n* = 5)	10	M:F = 9:1, 60–80 y	Advanced mandibular ridge resorption (Atwood class V and VI)	Yes	1 clinician	Swallowing NZ and Phonetic NZ	Tissue conditioning material	Tissue conditioning material & ZOE impression paste	Bilateral balanced articulation
Ladha et al. (2014) [23]	Randomized crossover clinical trial	1) old CV-SNZ-PNZ (*n* = 5) 2) old CV-PNZ-SNZ (*n* = 5)	10	M:F = 9:1, 60–80 y	Advanced mandibular ridge resorption (Atwood class V and VI)	Yes	1 clinician	Swallowing NZ and Phonetic NZ	Tissue conditioning material (ZOE impression paste for washing the impression)	Tissue conditioning material	Bilateral balanced articulation
Rehmann et al. (2016) [11]	Quasi-experiment (before and after)	Before (existing CV) & After (new NZ) (*n* = 21)	21	M:F = 1.1:1, mean=71 ± 19 y	Severely resorbed mandible^a^	Yes	1 prosthodontist perform clinical works	Modified NZ by Rehmann et al. (2012) [13]	Thermoplastic denture adhesive (Cushion Grip; Merck)	Thermoplastic denture adhesive	Bilateral balanced articulation
Rehmann et al. (2017) [12]	Quasi-experiment (before and after)	Before (new CV) & After (new NZ) (*n* = 21)	21	M:F = 1.1:1, mean=71 ± 19 y	Severely resorbed mandible^a^	Yes	1 prosthodontist perform clinical works	Modified NZ by Rehmann et al. (2012) [13]	Thermoplastic denture adhesive (Cushion Grip; Merck)	Thermoplastic denture adhesive	Bilateral balanced articulation
Geerts GAVM (2017) [28]	Randomized crossover clinical trial	1) NZ-CV (*n* = 17) 2) CV-NZ (*n* = 20)	37	M:F = 1:1.5, mean=62.3 ± 9.2 y (47–85)	N/A	Yes	1 prosthodontist for clinical and lab works	NZ by Cagna et al. (2009) [1]	Modeling plastic impression compound	ZOE impression paste	Lingualized balanced articulation
Al-Magaleh et al. (2019) [24]	Randomized crossover clinical trial	1) NZ-CV (*n* = 52) 2) CV-NZ (*n* = 52)	52	M:F = 1.3:1 mean=64.2 y	Class I max-man relation, adequate interarch distance, normal tongue behavior and size	No	2 calibrated clinicians, 1 lab technician	NZ	Polyvinylsiloxane	Tissue conditioning material	Bilateral balanced articulation

*N/A* not applicable, *CV* conventional denture fabrication technique, *M:F* male:female ratio, *max-man relation* maxilla-mandibular relationship, *NZ* neutral zone dentures or other denture space recording techniques, *ZOE* zinc oxide eugenol.

^a^Determined by mandibular bone height ≤20 mm in panoramic radiograph.

### Outcomes of interest

The treatment outcomes were assessed using the objective measures (Table 2) [9, 11, 12, 20–22], and the subjective measures based on the patient-reported outcomes (Table 3) [9, 11, 13, 16, 20, 21, 23, 24, 28]. The outcomes were evaluated following the use of each denture set [11–13, 16, 20–24, 28], except for Barrenas and Odman (1989) who conducted a single evaluation after the patients wore the final denture set [9]. They compared the outcomes between those who ended with the NZ and conventional dentures. The objective measures encompassed evaluations of denture-bearing mucosa, speech, mastication, and muscle function (Table 2). The objective findings varied, with most studies demonstrating equivalent outcomes for the NZ and conventional dentures [9, 11, 12, 21, 22]. However, one study indicated superior outcomes [21], while another demonstrated worse outcomes for the NZ denture [20].

**Table 2:** Objective outcomes regarding oral condition, masticatory function, and speech production (6 studies).

First author (Year)	Time of evaluation	Objective outcomes		Preferred denture
		Outcomes	Assessment	
Barrenas & Odman (1989) [9]	3–6 months post-insertion of the last denture set	Denture-bearing mucosa	Denture stomatitis	NZ = CV
Fahmy & Kharat (1990) [20]	2-week post-insertion of each denture set	Masticatory performance	Single sieve method of peanut particle size	CV
		Swallowing threshold	Peanut size up to swallowing	CV
Al-Magaleh et al. (2012) [21]	Immediate after insertion & 3-week post-insertion	Duration taken for recitation of Al-Fateha	Duration for continuous uninterrupted speech	NZ
		Acoustic analysis	Fricative and vowel durations of sounds using computer speech lab analyzer	NZ = CV
Ladha et al. (2013) [22]	2-month post-insertion of each denture set	Muscle activities at rest and during pursing, laughing, and pronouncing	buccinator, superior and inferior orbicularis oris	SNZ = PNZ = CV
Rehmann et al. (2016) [11]	4-week post-insertion	Masticatory function test	Six-level ordinal scale rating of raw carrot chewing	NZ = CV
Rehmann et al. (2017) [12]	4-week post-insertion	Speech sound production	Six-level ordinal scale rating	NZ = CV

= indicates similar outcome between the techniques, *CV* conventional denture fabrication technique, exist *CV* existing conventional denture, *NZ* neutral zone denture.

**Table 3:** Subjective outcomes regarding satisfaction, perception, and oral health-related quality of life (9 studies).

First author (Year)	Time of evaluation	Subjective outcomes		Preferred denture
		Outcome of interest	Measurement tools/methods	
Walsh & Walsh (1976) [16]	1-week	Patient satisfaction	Stability of LCD	NZ
Barrenas & Odman (1989) [9]	3–6 months after the last denture set	Patient selection of the preferred set	NV or CV	NZ
		Patient’s comments	NV or CV, considering - Esthetics - Comfort - Fit, adaptation - Food entrapment under denture, between denture and cheek	NZ (all aspects)
Fahmy & Kharat (1990) [20]	2-week post-insertion of each denture	Patient selection of the preferred set	NV or CV (considering comfort, retention, stability, speech, mastication)	NZ (Mastication: NZ = CV)
Al-Magaleh et al. (2012) [21]	Immediate after insertion & 3-week post-insertion	Patient satisfaction	Three-level ordinal scale: - Comfort - Retention, stability - Esthetics - Function	NZ
Rehmann et al. (2012) [13]	N/A	Patient perception	Perception on denture stability improvement in general, when chewing and speaking	NZ
Ladha et al. (2014) [23]	8-week post-insertion of each denture	Patient satisfaction	Five-level ordinal scale, considering 1) General, retention, stability, speech 2) Comfort, appearance, feeling part of body, ability to chew various food types, 3) Soreness under denture, 4) Food entrapment	1) NZ (SNZ = PNZ) [UCD stability: NZ = CV] 2) NZ (SNZ = PNZ) 3) NZ = CV 4) UCD: NZ = CV LCD: SNZ
		Patient selection of the preferred set	Considering - Comfort - Retention, stability - Speech - Mastication	SNZ [SNZ = PNZ for speak]
Rehmann et al. (2016) [11]	4-week post-insertion	Patient perception	Perception on denture stability improvement in general, when chewing and speaking	NZ
		OHRQoL	OHIP-G14	NZ
Geerts GAVM (2017) [28]	8-week after last recall visit of each denture set	OHRQoL	OHIP-20	NZ = CV
Al-Magaleh et al. (2019) [24]	6-week post-insertion of each denture (1-month wash-out period)	Patient satisfaction	Five-level ordinal scale: - In general - Own and other perception of appearance - Comfort - Retention, stability - Speech - Soft and hard food mastication	NZ (all aspects)

*LCD* mandibular complete denture, *UCD* maxillary complete denture, *CV* conventional denture fabrication technique, *NZ* neutral zone technique, *PNZ* phonetic neutral zone technique, *SNZ* swallowing neutral zone technique, *OHIP* Oral Health Impact Profile, *OHIP-G* German version of *OHIP*, *OHRQoL* Oral health-related quality of life.

The patient-reported outcomes comprised patient’s selection of a preferred set [9, 20, 23], patient satisfaction [9, 11, 21, 23, 24], and the OHRQoL (Table 3) [11, 28]. Compared with conventional dentures, the NZ dentures provide greater comfort [9, 20, 21, 24], enhanced denture stability and retention [9, 11, 20, 23, 24], reduced food trap underneath the denture [9, 23], improved appearance [9, 20, 23, 24], improved chewing efficiency [9, 24], better speech [11, 20, 24], and enhanced the OHRQoL [11]. NZ dentures were perceived as more integrated with the body compared to conventional dentures [23]. Following the experience of both denture sets, patients predominantly preferred the set fabricated using the NZ technique compared with those made with the conventional technique [9, 20]. Only Geerts GAVM (2017) found that both conventional and NZ dentures equally improved the OHRQoL of patients [28].

### Risk of bias assessment

**Fig. 2:**
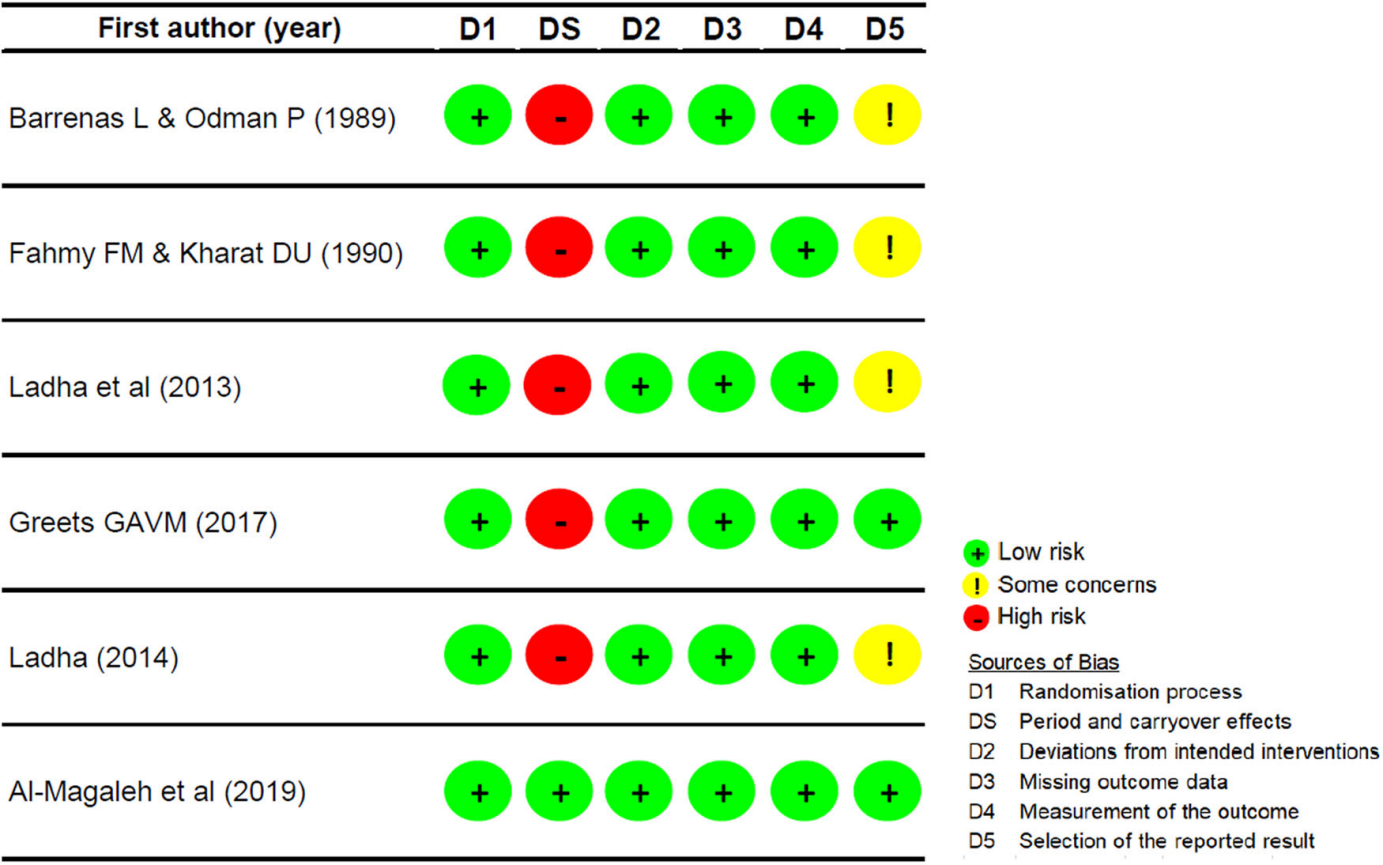
Risk of bias assessment of the randomized crossover experimental studies.

**Fig. 3:**
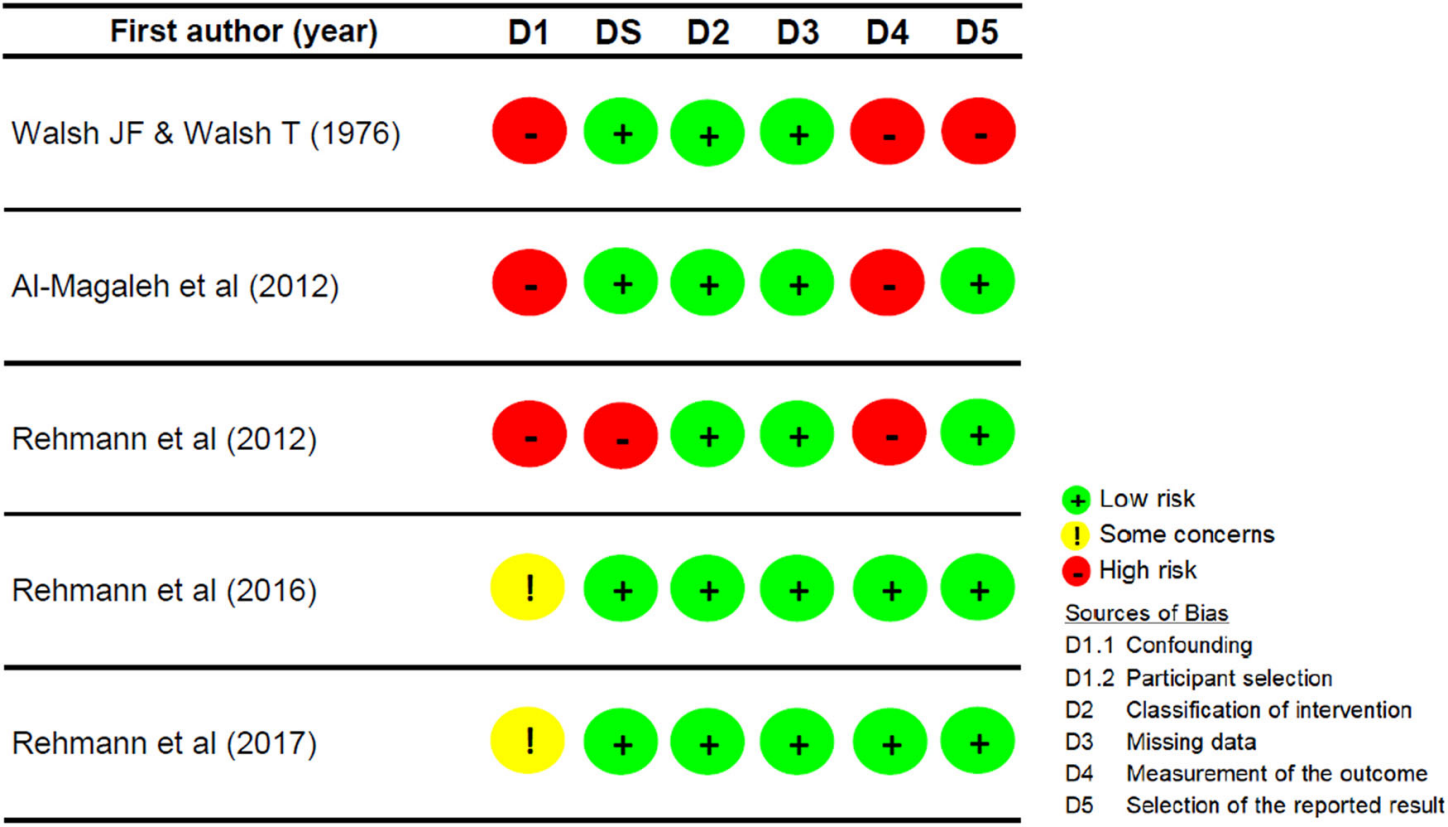
Risk of bias assessment of the non-randomized studies.

The results of the quality appraisal of the included studies are presented in Figs. 2 and 3. Among studies employing a crossover trial design, only Al-Magaleh et al. (2019) provided a one-month wash-out period (Fig. 2) [24]. On the contrary, the others did not incorporate a wash-out period when transitioning dentures to the other type, presenting a high risk of bias attributed to a potential carry-over effect [9, 20–23, 28]. The predominant source of bias in the included non-randomized studies was mostly due to confounding factors (Fig. 3) [11–13, 16, 21]. These confounders were associated with distinct participant characteristics, such as previous denture-wearing experience and the severity of the edentulous condition.

## Discussion

To the best of our knowledge, this systematic review is the first to examine patient-oriented outcomes following receiving CD treatment with the NZ technique compared with the conventional approach. Our findings indicate improved patient-report outcomes, including denture selection, higher satisfaction levels, and enhanced OHRQoL, for those utilizing NZ techniques. However, the objective measures, including speech, masticatory performance, and muscle activities varied across studies, with the majority reporting equivalent outcomes between NZ and conventional dentures.

Compared with the conventional approach, NZ dentures generally results in higher patient satisfaction and preference [9, 11, 13, 16, 20, 21, 23, 24]. After experiencing the two denture sets, patients typically favored NZ dentures compared with conventional ones [9, 20, 23]. They reported that NZ dentures offered superior comfort, denture retention, and stability. This is attributed to the fact that the polished surface of NZ dentures is designed to complement the contours and functions of the tongue, lips, and cheeks, both at rest and during oral function [20, 24]. Thus, the artificial teeth are positioned within the zone of muscle balance [24], enhancing speech and masticatory abilities. Furthermore, the polished surfaces and contoured borders contribute to fuller lips and cheeks, resulting in improved facial support and appearance [9, 20, 23]. However, it is noted that one included study reported better masticatory perception for conventional approach [20]. This could be due to the relatively short evaluation period of two-week post-insertion, which may not allow sufficient time for masticatory adaptation.

Minor disparities exist regarding the impact on the OHRQoL outcomes when comparing NZ dentures with the conventional dentures. One study reported greater OHRQoL improvement for NZ dentures [11], while another found similar improvement between conventional and NZ dentures [28]. This similar improvement between the two treatment approaches could be attributed to the fact that the participants in this study were patients who perceived a treatment need and were dissatisfied with their existing prostheses [28]. Furthermore, it is possible that the OHRQoL tool assesses the overall oral health condition, whereas satisfaction tools are specifically designed for a particular purpose, providing higher sensitivity to detect minor differences among treatments [24].

The findings related to objective measures varied across studies. Most included studies that did not find significant differences in objective measures between the two techniques, regarding muscle activity [22], the masticatory function test [11], and speech tests [12, 21]. The lack of difference in muscle activity between conventional and NZ dentures may be due to the fact that the post-insertion adaptation period was insufficient for peri-oral muscles to fully adapt to the new denture [22]. Additionally, sound production assessed by a speech test is influenced by factors beyond just the external surface, including the maxillary anterior tooth position, and palatal contour and thickness [12, 21]. The variations in objective measures could also be attributed to the time required for individual patient adaptation. Previous evidence suggests that a four-week period is generally considered optimal for adapting to a new CD [11, 12]. However, it is noted that older individuals may require an extended adaptation period beyond the typical four weeks [27]. In addition, mastication and speech production also depend on factors other than the impression technique. These factors are, for example, the assessment protocols and patient-related factors, such as muscle mass and strength, lip position, tongue size and position, and degree of mouth opening [3, 19, 21].

Comparing different NZ techniques, specifically the swallowing NZ and phonetic NZ approaches, similar muscle activities were observed through electromyography [22], and patient satisfaction levels [23]. In the phonetic NZ or piezography technique, patients are required to continuously pronounce words without swallowing until the impression material polymerizes. Conversely, for the swallowing NZ technique, patients are instructed to perform lip movements, such as lip pursing, sucking, and swallowing. Although one of the included studies found that swallowing NZ dentures might be a preferred choice over phonetic NZ dentures, the results should be interpreted cautiously due to the relatively small sample size in the study [23].

Considering other factors that would impact treatment outcome, different ages and sexes have no impact on patient satisfaction with NZ and conventional dentures [11, 12]. However, the mandibular residual ridge condition could potentially affect patient satisfaction in that those with a more resorbed ridge reported greater improvement for NZ compared with conventional denture [9, 24]. This is supported by a previous study that the NZ technique should be suitable for those with severe ridge resorption and not for general purposes because of its complexity, which is time-consuming [13]. Thus, the NZ technique is indicated for patients with severe mandibular resorbed alveolar ridges because it provides better retention and stability [1, 9].

Some potential biases should be noted for the included studies. First, only a few studies described a sample size calculation or power analysis to determine whether the number of participants was adequate to detect a clinically relevant treatment effect [24, 28]. Thus, the difference in treatment outcome between NZ and conventional dentures in some included studies may not have been detected and should be interpreted with caution due to their relatively low sample size without a sample size calculation [13, 22, 23]. Although a crossover clinical trial study design minimizes confounders occurring between patients that potentially affect the outcomes of interest, most of the studies had no wash-out period between NZ and conventional dentures [9, 20, 22, 23, 28]. This could be because of ethical concerns where the included patients were those who requested a new dental prosthesis due to dissatisfaction with their present denture [9, 22, 28]. Lastly, because a single operator provided denture treatment, the operator was not blinded to the intervention given to the patient during each crossover trial period [9, 20, 22, 23, 28]. However, due to a clearly defined treatment protocol, the treatment was less likely to deviate from the intended intervention, resulting in a low risk of bias.

The present study notes several clinical implications. The present systematic review demonstrates positive effects on maxillary and mandibular CDs fabricated by NZ techniques. For mandibular dentures, the NZ technique benefits severe mandibular ridge resorption because it provides optimal denture retention and stability [9, 23]. Although conventional maxillary CDs are generally stable, NZ maxillary dentures provide a better appearance due to the optimal contour of the polished surface [9, 23]. The reason for detecting change using subjective measures is because they are more sensitive to denture retention and stability changes than objective measures [2], which require time for patient adaptation. To evaluate the treatment outcome, subjective measures should be included in daily routine practice and clinical research, and those with a specific purpose may be required to detect changes based on a specific treatment.

This systematic review acknowledges certain limitations. Due to heterogeneities in the outcome of interest and assessment methods, the pooled estimates for meta-analysis could not be performed. Most of the included experimental studies involved a limited number of operators and a single laboratory technician conducting the clinical and laboratory work [9, 11, 12, 22–24, 28]. Consequently, the generalizability of the findings may be restricted to expert or experienced dentists and dental technicians where laboratory procedures can be more complex, such as techniques for preserving recorded denture space. Further investigations should explore the potential simplification of treatment procedures using digital technology in NZ denture fabrication. Observational studies could also investigate whether clinician experience impacts treatment outcomes due to technique sensitivity. Furthermore, there is a need for additional research on how previous denture experience and patient adaptation to new dentures may influence the objective outcomes of NZ treatment with an extended duration of denture use.

## Conclusions

Based on the findings from this systematic review, it can be concluded that NZ dentures generally better enhance patient-reported outcomes compared with conventional dentures. However, the impact of NZ dentures on objective outcomes compared with conventional dentures remains uncertain.

## Data Availability

Dataset generated during the current study is available upon request to the corresponding author.
